# A review of methods for detect human *Papillomavirus* infection

**DOI:** 10.1186/1743-422X-9-262

**Published:** 2012-11-06

**Authors:** André L P Abreu, Raquel P Souza, Fabrícia Gimenes, Marcia E L Consolaro

**Affiliations:** 1 Division of Clinical Cytology, Department of Clinical Analysis and Biomedicine, State University of Maringá, Av. Colombo 5790, 87020-900, Paraná, Brazil

**Keywords:** Human *Papillomavirus*, Detection methods, Biomarkers, Cervical cancer, Cervical lesions progression

## Abstract

Human *Papillomavirus* (HPV) is the most common sexually transmitted virus. Worldwide, the most common high-risk (HR)-HPV are -16/18, and approximately 70% of cervical cancers (CC) are due to infection by these genotypes. Persistent infection by HR-HPV is a necessary but not sufficient cause of this cancer, which develops over a long period through precursor lesions, which can be detected by cytological screening. Although this screening has decreased the incidence of CC, HPV-related cervical disease, including premalignant and malignant lesions, continues to be a major burden on health-care systems. Although not completely elucidated, the HPV-driven molecular mechanisms underlying the development of cervical lesions have provided a number of potential biomarkers for both diagnostic and prognostic use in the clinical management of women with HPV-related cervical disease, and these biomarkers can also be used to increase the positive predictive value of current screening methods. In addition, they can provide insights into the biology of HPV-induced cancer and thus lead to the development of nonsurgical therapies. Considering the importance of detecting HPV and related biomarkers, a variety of methods are being developed for these purposes. This review summarizes current knowledge of detection methods for HPV, and related biomarkers that can be used to discriminate lesions with a high risk of progression to CC.

## Introduction

Human *Papillomavirus* (HPV) is the most common sexually transmitted virus [1]. There are around 100 types of HPV, with different variations in their genetic and oncogenic potential [2]. Cervical cancer (CC) is caused by types of HPV that belong to a few phylogenetically related “high-risk” (HR) species (alpha-5, 6, 7, 9, 11) of the mucosotropic alpha genus [3,4]. The types found most frequently in CC (-16, -18, -31, -33, -35, -45, -52, -58) and four less-common types (-39, -51, -56, -59) were classified in Group 1. The remaining types of HPV in the HR alpha species were classified as “possibly carcinogenic” (Group 2. 2A: -68; 2B: -26, -30, -34, -53, -66, -67, -69, -70, -73, -82, -85, -97). Finally, HPV -6 and -11, which belong to the alpha-10 species, were “not classifiable as to their carcinogenicity in humans” (Group 3) [5] and were also described as “low risk” (LR) [6].

Worldwide, the most common HR-HPV are -16/18, and approximately 70% of CC are due to these genotypes. LR-HPV, principally -6/11, are predominantly involved in the development of genital warts [6]. CC is the second most common cancer in women worldwide, and is a major cause of morbidity and mortality [7]. Persistent infection with HR-HPV is a necessary but not sufficient cause of this cancer, which develops over a long period of time through precursor lesions, which can be detected by cytological screening. The majority of these lesions regress spontaneously without treatment. The challenge of CC screening is to detect the lesions that have a high risk of progression [8,9].

Although cervical cytology screening has decreased the incidence of CC, HPV-related cervical disease, including premalignant and malignant lesions, continues to represent a major burden on health-care systems. Some of the problems include the potential for either under- or overtreatment of women, due to low specificity of screening tests, as well as to significant variability in the diagnosis of cervical dysplastic lesions. Although not completely elucidated, the HPV-driven molecular mechanisms underlying the development of cervical lesions have provided a number of potential biomarkers for both diagnostic and prognostic use in the clinical management of these women, and have increased the positive predictive value of current screening methods [10].

Considering the importance of detection of HPV and related biomarkers, several methods are being developed for these purposes. This review summarizes current knowledge about detection methods for HPV and related biomarkers that can be used to discriminate lesions with a high risk of progression to CC.

### Molecular methods for HPV detection

**Figure 1:**
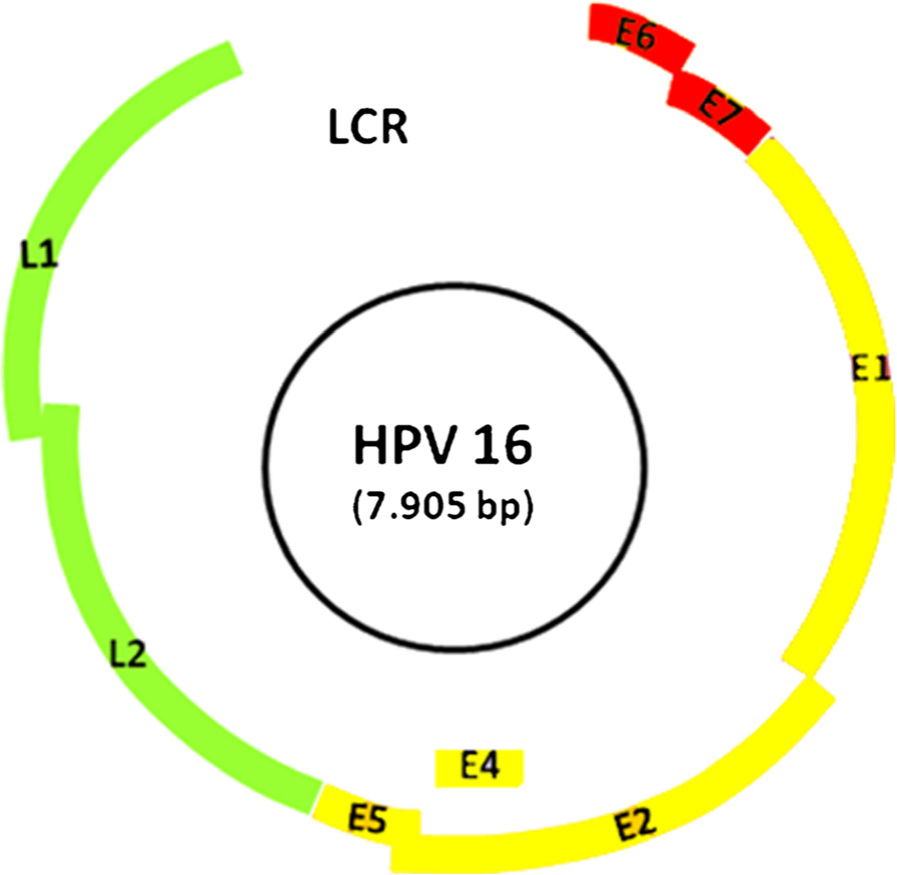
**Genome organization of HPV. **Location of the HPV major proteins. The HPV genome encodes early proteins with regulatory (E1 and E2) and transforming (E6 and E7) functions and two late capsid proteins (L1 and L2). Protein E4 has a largely unknown function and E5 is a hydrophobic protein that enhances cell immortalization. Adapted from Faridi R, Zahra A, Khan K, Idrees M. **Virol J**. 2011; 8:269.

HPV cannot be propagated in tissue culture, and therefore, in most cases its accurate identification relies on molecular biology techniques. With a double-stranded DNA genome of about 8000 base pairs (bp) and a well-known physical structure and gene organization, the tests of choice for detecting HPV in clinical specimens are based on nucleic probe technology [11] (Figure 1).

The six main possible clinical applications of HPV DNA testing are: (i) triage of women with equivocal or low-grade cytological abnormalities; (ii) follow-up of women with abnormal screening results who are negative at colposcopy/biopsy; (iii) prediction of the therapeutic outcome after treatment of cervical intraepithelial neoplasia (CIN); (iv) primary screening for HPV DNA testing, alone or in combination with a Pap smear, to detect cervical-cancer precursors [12]; (v) gain valuable information on the persistence of certain HPV types [13]; and (vi) investigations of regional and country-based prevalence of type-specific HPV, to provide baseline values against which the global impact of HPV vaccination can be assessed in the future [14].

Two HPV vaccines are commercially available, a bivalent (types -16/18) and quadrivalent (types -6, -11, -16 and 18) [15]. Vaccination against HPV-16/18 in particular can potentially prevent more than two-thirds of CC worldwide. Promising new broad-spectrum HPV vaccines are in development [16]. In addition, novel strategies based on the use of HPV DNA assays for primary cervical screening are increasingly recommended [14].

**Figure 2:**
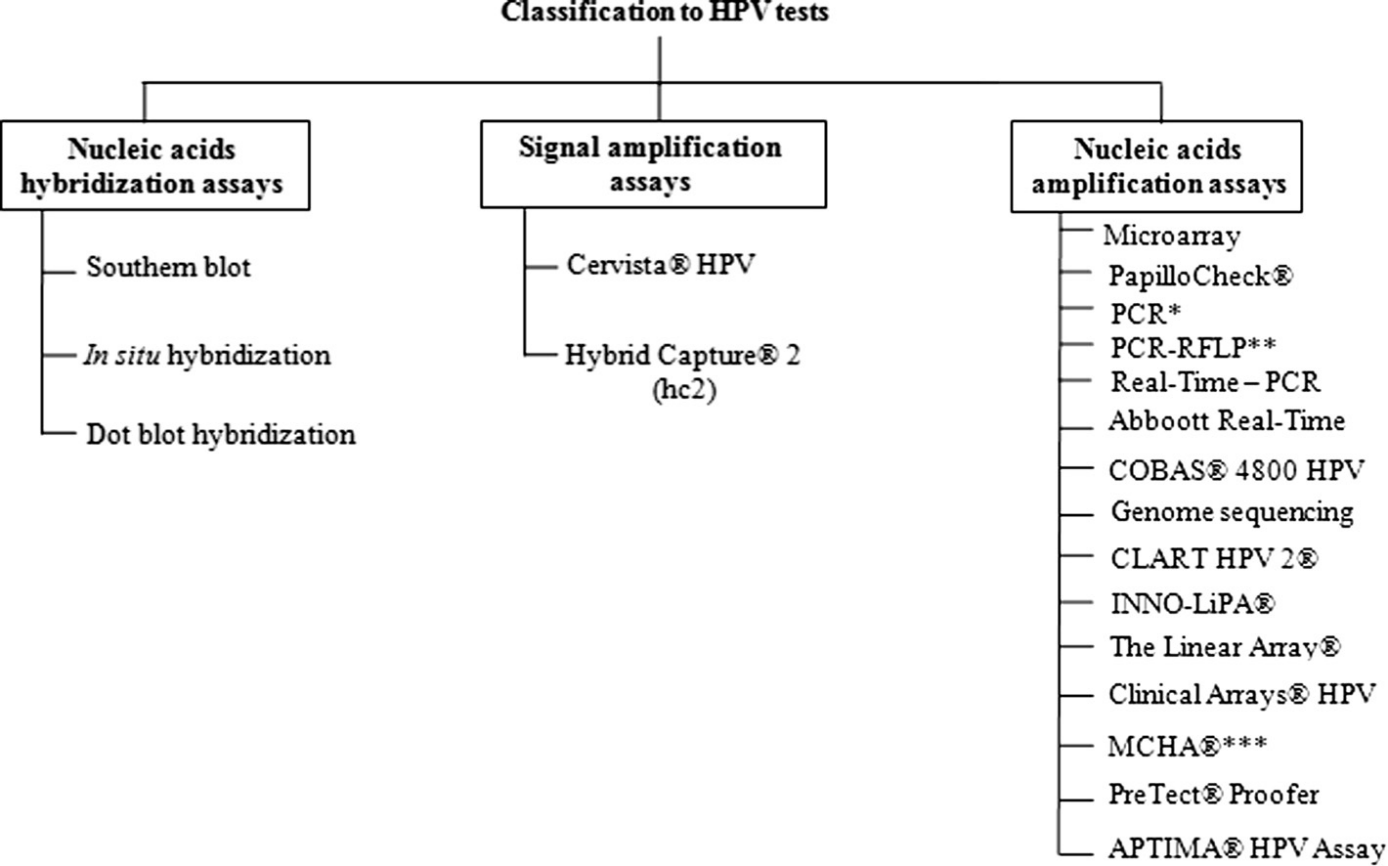
Schematic diagram of classification for HPV tests.

The presence of HPV can be inferred from morphological, serological and clinical findings [11,17]. However, HPV diagnosis relies on molecular-biology techniques that allow its accurate detection and typing [18]. At present, nucleic acid-hybridization assays, signal-amplification assays and nucleic-acid amplification are available (Figure 2) (Table 1).

**Table 1:** Benefits and weaknesses of the molecular methods for HPV detection

**Method**	**Benefits**	**Weaknesses**
**Nucleic acids hybridization assays**	Southern blot is gold standard for HPV genomic analysis Presence of HPV in association with morphology	Low sensitivity , time consuming, relatively large amounts of purified DNA
		Southern blot and hybridization cannot use degraded DNA
**Signal amplification assays**	Quantitative	Licensed and patented technologies
	FDA-approved test (hc2)	Wasn’t designed to genotyping individual
	Lower false-positive rate	
	High sensitivity to genotyping	
**Nucleic acids amplification assays**	Flexible technology (viral load and genotype)	Lower amplification signals of some HPV genotypes
	Very high sensitivity	Contamination with previously amplified material can lead to false positives
	Multiplex analysis	

HPV = Human *Papillomavirus*; FDA = Food and Drug Administration; hc2 = Hybrid Capture® 2; PCR = Polymerase Chain Reaction.

### Nucleic acid-hybridization assays

Initially, techniques such as Southern blotting, *in situ* hybridization, and dot-blot hybridization used radio-labeled nucleic acid hybridization assays to detect HPV infection in cervical samples. Although these techniques generated high-quality information, the disadvantages of these direct-probe approaches include low sensitivity, the need for relatively large amounts of purified DNA, and time-consuming procedures [11].

### Signal-amplification assays

The Digene® HPV test using Hybrid Capture® 2 (hc2) technology, and the Cervista® HPV HR assay are the only methods that currently have FDA (Food and Drug Administration) approval for diagnostic testing in the United States [19].

The Hybrid Capture® 2 system (hc2, Digene Corp., USA) is a non-radioactive signal-amplification method based on the hybridization of the target HPV-DNA to labeled RNA probes in solution [20]. This test detects 13 HR-HPV types (-16,-18,-31,-33,-35,-39,-45,-51,-52,-56,-58,-59 and -68) or 5 LR-types (-6, -11, -42, -43, and -44) [19].

This assay distinguishes between HR and LR groups, but was not designed for genotyping single HPV [19]. This is a significant finding, since with persistent infection the risk of a precancerous lesion is between 10 and 15% with HPV types -16/18, and below 3% for all other HR types combined. Therefore, HPV genotyping is very important to identify single oncogenic HPV types [21] and to provide more information regarding risk-stratification as well as persistence of infection [22,23].

The Cervista® HPV (Hologic, Inc., Marlborough, MA, USA) detects the presence of 14 HR-HPV types, consisting of -16,-18,-31,-33,-35,-39,-45,-51,-52,-56,-58,-59,-66 and -68 [19]. This assay also utilizes a signal-amplification method for the detection of specific nucleic acids.

In comparison with hc2, the Cervista® assay demonstrated 100% sensitivity in the detection of CIN III and 98% sensitivity in the detection of CIN II [24]. In addition, this assay showed a lower false-positive rate, and high sensitivity and specificity to genotyping HPV -16/18 [25,26].

### Nucleic acid-amplification methods

#### Microarray analysis

This method uses probe amplification, the PCR (**Polymerase chain reaction)** product is hybridized onto a chip, and after a washing step, hybridized signals are visualized with a DNA chip scanner [27]. The microarray-based automated techniques allow for parallel analysis of multiple DNA samples. At present, the two major applications of DNA microarrays are gene-expression profiling and mutation analysis [28].

Some studies have demonstrated that DNA microarray analysis coupled with PCR can be successfully applied to detection and genotyping of the HPV. The HPV DNA chip showed higher sensitivity and specificity than gel electrophoresis, and in some cases produced better results than direct DNA sequencing [29,30].

#### PapilloCheck®

This assay detects and genotypes 24 HPV types in a single reaction (HPV -6, -11, -16, -18, -31, -33, -35, -39, -40, -42, -43, -44, -45, -51, -52, -53, -55, -56, -58, -59, -66, -68, -70, -73, and -82). The assay uses a multiplex PCR with fluorescent primers to amplify a 350 bp fragment of the E1 gene of HPV, comprising 28 probes, each in 5 replicate spots fixed on a DNA chip. Co-amplification of the human *ADAT1* gene is used as internal control. The hybridization is performed on a microarray chip, which is automatically scanned and analyzed using the CheckScanner™ at both 532 and 635 nm, and the Check-Report™ software, respectively [31,32].

The main advantages of the PapilloCheck***®*** assay (Greiner Bio-One GmbH, Frickenhausen, Germany) is HR/LR-HPV identification, and detection of multiple infections, and may be considered a reliable screening test [30]. However, this assay does not amplify HPV -35 and -53, the cost is still relatively high, and it requires specific apparatus [33-35].

#### Polymerase chain reaction (PCR)

The PCR-based techniques are highly sensitive, specific, and widely used. In a conventional PCR, the thermostable DNA polymerase recognizes and extends a pair of oligonucleotide primers that flank the region of interest. In the final process, the PCR can generate one billion copies from a single double-stranded DNA molecule after 30 cycles of amplification [36].

The HPV-PCR protocols use consensus primers such as PGMY09/PGMY1 and GP5+/GP6+, which allow amplification of a large number of HPV genotypes in a single reaction. The primers target conserved regions of the HPV genome, such as the L1 capsid gene [37]. After amplification, the HPV genotypes can be determined separately, using techniques such as restriction-fragment length polymorphism (RFLP), linear probe assays, direct sequencing, or genotype-specific primers [38]. Some researchers have used a type-specific PCR, with primers that amplify the long control region L1 and E6/E7 [39].

These PCR techniques also have some drawbacks, mainly in competition for reagents, leading to false negative results for multiple type infections that are contained in samples at lower copy numbers. Because of this problem, the PCR method may not detect all the HPV genotypes that are present in the sample. Another downside is that multiple infections are not uncommon [29]. Amplification of samples containing DNA from more than one HPV genotype can lead to a much stronger amplification of one of the sequences present, which would complicate the detection of all genotypes in a sample with multiple infections. Sometimes, additional, labor-intensive procedures, such as sequencing or type-specific PCR, are required [38].

#### PCR-RFLP

Genotyping by PCR-RFLP allows the HPV to be typed, and is easier and less expensive than sequencing [40]. The method is simple and robust, does not require sophisticated equipment, and is particularly suited to settings in which financial resources are limited [38,41]. PCR-RFLP shows good discriminatory power by differentiating the virus in HR or LR, and it is possible to identify single or multiple infections. In this technique, the amplified DNA is digested by restriction enzymes, resulting in DNA fragments of various lengths. The commonest restriction enzymes are *Bam*HI, *Dd6e*I, *Hae*III, *Hin*fI, *Pst*I and *Rsa*I [42]. However, Santiago et al. used a single restriction enzyme, *Hpy*CH4V, to detect 21 HR- and 31 LR-HPV genotypes [43].

#### Real-time PCR

This assay is a reliable, sensitive, and specific diagnostic tool for detection and genotyping of targeted HPV genotypes in tissue specimens [44] and cellular samples. The advantages of this method are: (i) ability to detect viral load; (ii) with the use of different fluorochromes that emit fluorescence, as the PCR reaction proceeds, the reactions can be performed in multiples and can amplify different nucleic-acid targets; (iii) nucleic acids can be detected even in very small concentrations, using a 7-log dynamic range to extrapolate the viral load/concentration over the standard curve; and finally, (iv) it is extremely reproducible, rapid, and applicable to clinical samples [45].

#### Abbott real-time PCR

The Abbott Real-Time HR-HPV test is a novel assay based on concurrent individual genotyping for HPV-16 /18 and pooled detection of 12 HPV genotypes: -31, -33, -35, -39, -45, -51, -52, -56, -58, -59, -66 and -68 [44].

#### COBAS® 4800 HPV test

This test features automated sample preparation combined with Real-Time PCR technology to detect 14 HR-HPV. The PCR amplification and detection occur in a single tube, (i) HPV -16, (ii) HPV -18, (iii) 12 HR (-31, -33, -35, -39, -45, -51, -52, -56, -58, -59, -66, and -68) as a pool, and (iv) β-globin as the control for extraction and amplification adequacy [46].

The agreement between COBAS***®*** 4800 (Roche Molecular Systems, Pleasanton, CA, USA) and Real-Time PCR was strong in a study that determined the reproducibility, involving a sequence of several consecutive steps, both intra- and interlaboratory [46]. The assay is easy to use because it is adapted for primary specimens, and the results can be obtained approximately 4h after the sample is received. COBAS***®*** 4800 fulfills all requirements as defined in the international guidelines to consider it clinically validated for screening, and is reliable in the detection of HR-HPV [47]. This test has been clinically validated for ASC-US triage [48].

#### HPV genome sequencing

The dideoxy chain-termination technique (Sanger technique) was first described for genome sequencing more than three decades ago [49]. Fluorescently labeled nucleotides were incorporated into Sanger sequencing, and advances have led to increasing expansion and development of high-quality, thorough sequencing [50,51]. However, it has not been validated for clinical use.

Similar to dideoxy sequencing methods, pyrosequencing is applicable to any source of DNA or RNA that can be amplified by PCR (blood, saliva, cell line, plasma, serum, tissue, formalin-fixed paraffin-embedded samples, and whole genome-amplified DNA). The method is based on the detection of the pyrophosphate released during DNA synthesis, and has many advantages over dideoxy sequencing for a wide range of applications that require short-to medium-sequence stretches. The primary advantage is simplicity: the readout sequence itself is obtained, rather than a fluorescent signal that must be converted to a sequence. Second, it is faster and less expensive: savings result from its sequence-by-synthesis process where a DNA sequence is read in real time, and it is synthesized by addition of inexpensive, unlabeled nucleotides; and finally, the method is uniquely quantitative [52].

#### CLART® human papillomavirus 2

The *CLART*® *Human Papillomavirus 2* (Genomica, Madrid, Spain) methodology uses biotinylated primers that amplify a 450 bp fragment within the HPV L1 region. Co-amplification of an 892 bp region of the FTR gene and a 1.202 bp fragment of a transformed plasmid provides a control to ensure DNA extraction adequacy and PCR efficiency. Amplicons are detected by hybridization in a low-density microarray containing triplicate DNA probes specific for 35 HPV (-6, -11, -16, -18, -26, -31, -33, -35, -39, -40, -42, -43, -44, -45, -51, -52, -53, -54, -56, -58, -59, -61, -62, -66, -68, -70, -71, -72, -73, -81, -82, -83, -84, -85 and -89). Semi-quantitative results can be obtained in an automatic reader with highly comparable outcomes, showing excellent sensitivity, specificity, and reproducibility [31].

#### INNO-LiPA

This assay genotypes all 14 HPV that are covered by Real-Time [44]. INNO-LiPA (LiPA HBV GT; Innogenetics N.V., Ghent, Belgium) is based on the co-amplification of the 65 bp region of the HPV L1 gene and the 270 bp of the human HLA-DP1 gene using SPF10 biotinylated primers, followed by genotyping [53,54]. Some carcinogenic genotypes such as HPV-35, -39, -52, -56 and -66 were not covered by this method, and it was found to be the least effective genotyping for HPV-42 and -59 [33].

Although the majority of nucleic-acid amplification methods can reliably detect HPV in cervical-swab specimens, only a few, including Real-Time PCR, are potentially suitable for archival clinical specimens, since they target a relatively small portion of the HPV genome (less than 160 bp). Therefore, the observed differences in internal control amplification efficacy between Real-Time and INNO-LiPA can be attributed most reasonably to the differences in target amplicon length: 136 bp vs. 270 bp, respectively [44]. This kit can be also used on samples taken with swabs, brushes, tampons, and lavage [55,56].

#### The Linear array®

The Linear Array***®*** HPV Genotyping (Roche Molecular Diagnostics, Pleasanton, CA, USA) is a PCR-based assay coupled with a reverse line blot hybridization. This assay allows the discrimination of 36 HPV, including 15 HR (-16, -18, -31, -33, -35, -39, -45, -51, -52, -56, -58, -59, -68, -73 and -82), 3 probable HR (-26, -53 and -66), 10 LR (-6, -11, -40, -42, -54, -61, -70,-72, -81 and -CP6108) and 9 genotypes for which the risk is still undetermined (-55, -62, -64, -67, -69, -71, -83, -84 and -IS39) [57].

The test uses biotinylated PGMY09/11 primers to amplify a 450 bp fragment within the polymorphic L1 region of the HPV genome. Co-amplification of the 268 bp region of the human β-globin gene provides a control to ensure DNA extraction adequacy and PCR efficiency. The hybridization and detection of the amplified product are performed with the Auto-LIPA™ instrument (Innogenetics, Ghent, Belgium), which can process up to 30 strips simultaneously in a perfectly standardized fashion. Colored signals on the strips are read by the naked eye and interpreted according to the Linear Array***®*** reference guide. Equivocal results can be obtained for HPV-52 when -33, -35 or -58 are also present, because it is detected through a cross-hybridization probe for these 4 HPV types. An additional, specific probe is present on the strip to confirm the detection of HPV -33, -35 and -58, but not of HPV -52 [34,58].

#### Clinical arrays® HPV

This kit (Genomica SAU, Madrid, Spain) allows the detection and genotyping of HPV. The DNA extraction method is a modified procedure using absorption columns. The kit employs biotinylated primers to define a sequence of 451 nucleotides within the polymorphic L1 region of the HPV genome. A human cystic-fibrosis transmembrane conductance regulator (CFTR) gene and control plasmids are used to order to check both the PCR procedure and the integrity of the DNA [23].

This also allows the detection of the 35 genotypes that are individually associated with HR- (-16, -18, -26,-31, -33, -35, -39, -45, -51, -52, -53, -56, -58, -59, -66, -68, -70, -73, -82 and -85) or LR-HPV (-6, -11, -40, -42, -43, -44, -54, -61, -62, -71, -72, -81, -83, -84 and -89). It is possible to identify simple infections or co-infections [23,59].

#### Microplate colorimetric hybridization assay (MCHA)

The MCHA (Boehringer Mannheim, Germany) is a method for identifying six HR-HPV (-16, -18, -31, -33, -39 and -45) and is based on the amplification by PCR of the 150 bp fragment within the L1 region by consensus primers GP5+/6+, followed by colorimetric hybridization to six type-specific probes on microwell plates (Immobilizer™ Amino Surface, Nunc, Roskilde, Denmark) [35].

The MCHA showed very good agreement with PapilloCheck® for HPV-31, -33, -45 and higher sensitivity in identifying HPV -16/18, but poor agreement for -39. To improve MCHA for detection of other genotypes, probes for HPV-35, -52, -56 and -58 should be included [35].

#### HPV-mRNA detection

E6 and E7 are the main genes responsible for cell transformation mediated by HR-HPV, and they modulate the activities of cellular proteins that regulate the cell cycle [60]. Thus, the presence of E6/E7 can be a specific marker for diagnosing precancerous lesions by HPV [61]. For this reason, the search for transcripts of E6/E7 could increase the specificity and sensitivity of the tests in screening for cervical lesions that have a greater chance of progressing, compared with a simple detection of HPV-DNA [11,19,62].

The main techniques used to detect mRNA for E6/E7 oncogenes are two commercial assays: PreTect® Proofer and APTIMA® HPV Assay [63]. The chemistry is based on transcription-mediated amplification of full-length E6/E7 transcripts preempted by target capture.

The Pretect® HPV-Proofer assay (NorChip AS, Klokkarstua, Norway) detects E6/E7 mRNA from five HR-HPV (-16, -18, -31,-33, and -45). Clinical studies have shown high sensitivity. This assay is based on Real-Time multiplex PCR and is more specific than HPV-PCR for the detection of underlying HSIL (high-grade squamous intraepithelial lesions) [19].

The APTIMA® HPV assay (Gen-Probe, San Diego, CA, USA) detects HPV E6/E7 mRNA of the 14 HR (-16, -18, -31, -33, -35, -39, -45, -51, -52, -56, -58, -59, -66, and -68), which provides better sensitivity than the Proofer test, which detects only 5 HR-HPV [62]. This assay has several advantages over the other HPV tests, including: (i) detects HPV E6/E7 mRNA, which may be a better marker of advanced disease than hc2; (ii) the limit of detection is lower than the limits reported for other tests; (iii) it does not cross-react with LR-HPV types tested in the current study; and (iv) it is compatible with a fully automated processing system [19,62].

In addition, the oncoprotein E6 activates telomerase reverse transcriptase (TERT) expression and causes cellular immortalization [64]. Telomerase consists of several subunits, including a structural RNA component (hTR) that serves as a model during telomere elongation, and a catalytic subunit (hTERT) that has reverse transcriptase activity [65]. High levels of telomerase in tumor cells invariably result from deregulated hTERT expression, and can be detected by quantitative Real-Time PCR [66]. Also, studies have indicated that elevated hTERT expression is a frequent event during cervical carcinogenesis, and may be a valuable marker for progressive cervical lesions [67,68].

### HPV viral load quantification and integration

#### HPV-DNA viral load

The association between the HPV viral load and cervical lesions with malignant potential remains unclear. Many studies of HPV load address the utility of predicting the progression or severity of disease [69-72]. Lowe et al. demonstrated differences in load between CIN I and CIN II and between normal and all CIN, but not between CIN II and CIN III [72]. Thus, the results from load histology and cytology are in agreement. Lowe et al. also reported that the viral load declines in response to therapy, and provides an acceptable alternative for decisions to pursue further clinical trials [72]. However, other studies have shown that viral load assessment had no added value over cytology, and that testing for high load levels may not be clinically useful, except in the case of HPV -16 [70,71,73].

HPV viral load can be determined by Real-Time PCR techniques. These techniques have been used for semi-quantification in clinical samples, which can be also determined by hc2 [74].

#### HPV-DNA integration

HPV-DNA is usually present in extrachromosomal or episomal form in beginning cervical precursor lesions. Integration of viral DNA frequently occurs in HSIL and CC, and these lesions may often contain episomal and integrated HPV-DNA at the same time [75]. During HPV-DNA integration into the host cell, the viral genome usually breaks at E1 and/or E2 open reading frames (ORFs), whereas the E6/E7 ORFs and long control region remain intact [76,77]. Loss of the E2 gene function results in uncontrolled and increased expression of the oncogenic proteins E6 and E7. A high copy number of HPV-DNA directly contributes to HPV-DNA integration and increased expression of E6 and E7 [75], and is related to persistent HPV infection [74].

The viral integration is a very early event, it occurs earlier than the onset of morphological changes. Molecular events precede morphological features leading to malignancy, and that integration does not always temporally coincide with a high grade lesion. It is also possible that viral integration is not necessarily always followed by immediate viral E1 and/or E2 expression [78,79].

The main methods used for HPV integration detection are PCR, fluorescence *in situ* hybridization, and Real-Time PCR. The latter allows calculation of the ratio between the levels of E2 and E6/E7 HPV genes. When there is HPV integration, the viral genome shows a 1:1 ratio between the E2 and E6/E7 genes [74].

Frequently, HPV integration by PCR uses E2-type-specific primers in the HPV -16, -18 and/or E1-type-specific primers in the HPV -16 [77,80]. The PCR method is simple and easy to use, but it cannot be used to determine the site of integration or to distinguish between extrachromosomal or episomal forms, only the pure integrated form [81]. Fluorescence *in-situ* hybridization uses the locus-specific fluorescence probes TERC (human telomerase gene-region 3q26) and *MYC* gene (region 8q24) in cervical cytology specimens, and can be used to supplement the evaluation of the integration of HPV by PCR [82].

## Conclusions

CC develops over a long period, through precursor lesions that may regress spontaneously without treatment. The challenge of CC cytological screening is to detect the lesions that have a high risk of progression. Consequently, various biomarkers associated with the risk of progression of this cancer have been investigated, and most are associated with HR-HPV. Molecular techniques are most commonly used for HPV testing, and are the gold standard for diagnosing this viral infection. Some of these methods may also be used for investigations of regional and country-based type-specific HPV prevalence.

Cell infection by HPV is shown by changes in function or in host gene expression, and the detection of these changes may play a major role in the screening and follow-up of infected patients. HPV-DNA viral load quantification and integration, and E6/E7 expression are promising biomarkers that can predict the progression of lesions to CC, thus increasing the sensitivity of cytological screening. To date, there is no one ideal biomarker; however, overall, the combination of biomarkers can contribute to early determination of CC, which can be used to increase the positive predictive value of current screening methods. Therefore, this review summarizes current knowledge about detection methods for HPV and related biomarkers. In spite of their value, molecular techniques still must become more rapid, automated, and low-cost to be of practical use in low-income populations and countries.

## Abbreviations

HPV: Human *Papillomavirus*; CC: Cervical cancer; HR: High-risk; LR: Low-risk; bp: Base pairs; CIN: Cervical intraepithelial neoplasia; hc2: Hybrid Capture 2; PCR: Polymerase chain reaction; RFLP: Restriction fragment length polymorphisms; ASC-US: Atypical Squamous cells of undetermined significanceADAT1, Human Adenosine Deaminase tRNA-Specific 1; MCHA: Microplate Colorimetric Hybridization Assay; HSIL: High-grade squamous intraepithelial lesions; TERT: Telomerase reverse transcriptase; FTR: Formylmethanofuran: tetrahydromethanopterin formyltransferase; ORFs: Open reading frames; TERC: Human telomerase gene.

## Competing interests

The authors declare that they have no competing interests.

## Author’s contributions

ALPA and FG searched the literature, organized the data. MELC, ALPA and FG wrote the manuscript. RPS helped in literature review and participated in its design. ALPA has been involved in revising the manuscript critically for important intellectual content. MELC revised the manuscript, helped to provide information and suggestion. MELC is the corresponding author. All the authors read and approved the final of the manuscript.

## Author’s information

**André Luelsdorf Pimenta de Abreu** graduated in Nurse at the Cesumar in 2009. He is currently doctoral student in Clinical Citology at the State University of Maringa. He has research interests in the HPV and biomarkers. **Raquel Pantarotto Souza** graduated in Pharmacy-Biochemistry at the State University of Maringa in 2010. She is currently master student the same university. Her scientific activity has been mainly intent in cervical cancer and HPV. **Fabrícia Gimenes** graduated in Biological Science at the State University of Maringá in 2004. In 2006 she became master in genetics and in 2010 she became doctor in Molecular and Biology Cell the same university. Since 2010 she has been post-doctoral student in Clinical Citology. She has research interests in the cervical cancer, HPV and biomarkers. **Marcia Edilaine Lopes Consolaro** Graduated in Pharmacy-Biochemistry at the State University of Maringá, Master and PhD degree in Biological Sciences (Cell Biology), since 1996 she is Assistant Professor of Clinical Cytology at the same university. Her teaching activity is currently held in the official courses of pharmacy and biomedicine, had experience in Pharmacy, enabling Clinical Analysis with Emphasis on Clinical Cytology. Her scientific activity has been mainly intent on issues related to HPV, cervical cancer, vaginal candidiasis, sexually transmitted diseases and herbal agents with properties against STDs. Coordinator of the Graduate Program in Biosciences Applied to Pharmacy.
